# Competing endogenous RNA network analysis of the molecular mechanisms of ischemic stroke

**DOI:** 10.1186/s12864-023-09163-1

**Published:** 2023-02-08

**Authors:** Jian-Min Chen, Xiao-Lu Li, Yang Ye, Sen-Ming Xu, Qing-Fa Chen, Jian-Wen Xu

**Affiliations:** 1 Department of Rehabilitation Medicine, The First Affiliated Hospital of Fujian Medical University, Fuzhou, Fujian China; 2 Department of Rehabilitation Medicine, The First Affiliated Hospital of Guangxi Medical University, Nanning, Guangxi China; 3 Department of Rehabilitation, Fujian Medical University Union Hospital, Fuzhou, Fujian China

**Keywords:** Ischemic stroke, Network analysis, Inflammatory response, Ferroptosis, Biomarker

## Abstract

**Background:**

Ischemic stroke (IS) is a serious neurological disease that largely results in long-term disability and death. Extensive evidence has indicated that the activation of inflammation and ferroptosis significantly contribute to the development of IS pathology. However, the underlying molecular mechanism remains unclear. In this study, we aimed to identify potential biomarkers associated with IS through the construction of a competing endogenous RNA (ceRNA) network and to investigate the possible inflammatory and ferroptosis-related molecular mechanisms.

**Results:**

We identified 178 differentially expressed target messenger RNAs (DETmRNAs) associated with IS. As revealed through enrichment analysis, the DEmRNAs were mainly enriched in the inflammatory signaling pathways and also related to ferroptosis mechanism. The CIBERSORT algorithm showed immune infiltration landscapes in which the naïve B cells, naïve T cells, and monocytes had statistically different numbers in the cerebral infarction group compared with the control group. A ceRNA network was constructed in this study involving 44 long non-coding RNAs (lncRNAs), 15 microRNAs (miRNAs), and 160 messenger RNAs (mRNAs). We used the receiver operating characteristic (ROC) analysis to identify three miRNAs (miR-103a-3p, miR-140-3p, and miR-17-5p), one mRNA (TLR4), and one lncRNA (NEAT1) as the potential key biomarkers of the ceRNA network. The key mRNA and lncRNA were shown to be highly related to the ferroptosis mechanism of IS. The expression of these key biomarkers was also further validated by a method of quantitative real-time polymerase chain reaction in SH-SY5Y cells, and the validated results were consistent with the findings predicted by bioinformatics.

**Conclusion:**

Our results suggest that the ceRNA network may exert an important role in the inflammatory and ferroptosis molecular mechanisms of IS, providing new insight into therapeutic IS targets.

**Supplementary Information:**

The online version contains supplementary material available at 10.1186/s12864-023-09163-1.

## Introduction

Globally, stroke remained the second-leading cause of death and the third-leading cause of long-term disability [1, 2]. Statistically, about 17 million stroke cases occur worldwide each year [3], of which 85–90% are ischemic strokes (IS) [4]. Three of four survivors suffer from neurological defect-related sequelae, such as hemiplegia, cognitive impairments, or dysphasia, significantly affecting their health and quality of life and creating heavy burdens for families and society in general [5, 6]. Patients usually require long-term rehabilitation after the acute phase and need to receive continuous community support and nursing home care, thereby implying heavy societal and familial burdens [3].

Despite advances in understandings of the causes and treatments of ischemic stroke (IS), its exact molecular mechanisms are not yet entirely clear. Thus, a better understanding of the pathological processes of IS may provide novel therapeutic targets and methods for IS, thus improving prognosis [7]. Mounting evidence has suggested that inflammation may play a critical role in the progression of IS [8, 9]. Within a few hours of IS, interleukin-1 and tumor necrosis factor are elevated in peripheral blood leukocytes [7]. Multiple immune cells infiltrate the ischemic parenchyma from peripheral circulation via the broken blood-brain barrier to trigger innate and adaptive immune responses, inducing inflammatory or anti-inflammatory responses via distinct pathways [10, 11]. Furthermore, ferroptosis has also been proven to be closely related to the progress of cerebral stroke [12] Ferroptosis is a newly recognized type of regulated cell death defined by intracellular iron overload and distinct biological features [13]. Symbols of ferroptosis have been observed in animal models of IS including overloaded iron, shrunken mitochondria, accumulated lipid peroxidation, and upregulated reactive oxygen species (ROS) [14]. Preliminary evidence has revealed that ferroptosis deteriorates ischemia-induced brain damage, while the administration of specific ferroptosis inhibitors can effectively reverse induced damage [15]. Therefore, a comprehensive analysis of the ferroptosis genes in IS might provide new targets for stroke therapy [16].

As an important class of pervasive genes, several studies have found that non-coding RNAs (ncRNAs) play a key role in IS-induced neuroinflammation and iron overload [16–18]. MicroRNAs (MiRNAs) are 18–25-nucleotide-length noncoding RNA molecules that can regulate gene expression by destabilizing messenger RNA (mRNA) transcripts and inhibiting their shift into proteins [3, 19]. Li et al. demonstrated that upregulation of miR-103a-3p can alleviate inflammation, suppress cerebral ischemia-reperfusion injury, and exert neuroprotective effects [19]. Gamdzyk et al. showed that activating miR-17-5p transcription can inhibit inflammatory mediators in an embolic stroke model in mice [14]. In addition, Zhang et al. suggested that miRNA-27a agonist significantly aggravated ferroptosis and reduced neurological function scores in the permanent middle cerebral artery occlusion rat model [20].

Long non-coding RNAs (lncRNAs) are also a kind of non-coding RNA with more than 200 nucleotides in length that regulate target gene expression by interacting directly with mRNAs as a transcriptional regulator, or by acting as competing endogenous RNAs (ceRNAs), for miRNAs [21, 22] and have been implicated in the development of IS [3, 23]. For example, the knockdown of NEAT1 can significantly alleviate oxygen and glucose deprivation/reoxygenation (OGD/R) injury in neuronal cells by inhibiting an inflammatory response via the miR-374a 5p/NFAT5 axis [24]. Silencing PVT1 can reduce infarct size and suppress ferroptosis in brain ischemia/reperfusion mice models through overexpression of miR-214-mediated TFR1 and TP53 expression [16].

At present, the ceRNA axis has become a research hot spot, and the interactions among lncRNAs, miRNAs, and mRNAs may provide valuable insight into the molecular mechanisms of IS and present novel biomarkers or targets for rehabilitation treatment [1]. In the present study, mRNA, lncRNA, and miRNA expression profiles from IS and control samples were collected from the Gene Expression Omnibus (GEO) repository and used to establish ceRNA networks in IS. Following a comprehensive analysis, we determined the key mRNAs, miRNAs, and mRNAs in IS to plot the receiver operating characteristic (ROC) curve to explore their diagnostic validity [11]. We identified one lncRNA, three miRNAs, and one 1mRNA as potential biomarkers. We further explored the potential underlying mechanisms of the inflammatory response and ferroptosis on IS.

## Material and methods

**Fig. 1:**
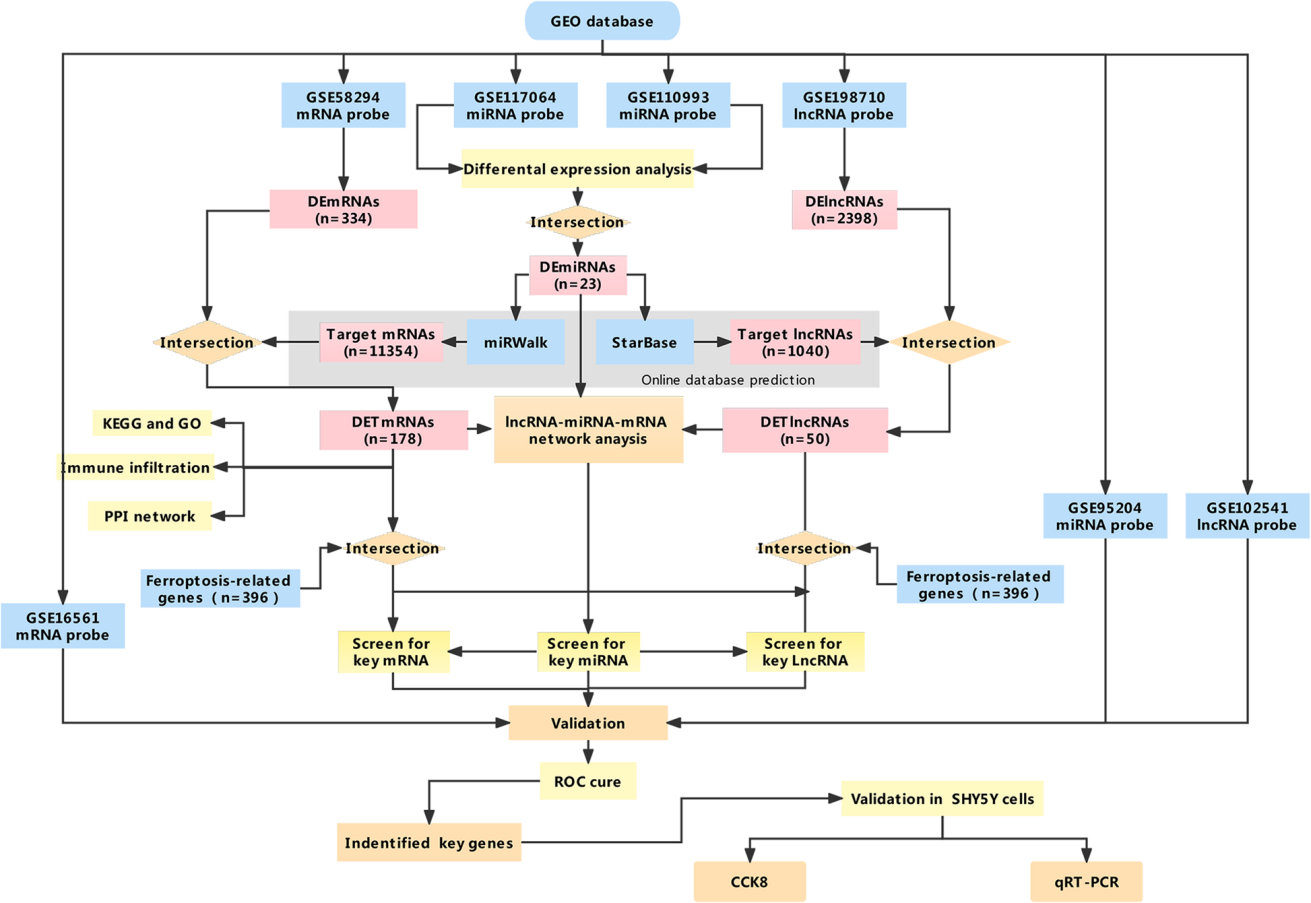
Flow chart of overall analysis

Expression profile data associated with stroke were obtained from the GEO database (https://www.ncbi.nlm.nih.gov/geo/), which is a public functional genomics data repository. IS-related datasets were retrieved using the keyword “stroke of *Homo sapiens*” (organisms). We included the gene expression profiling of the whole or peripheral blood of IS patients or control samples. Four datasets with reliable sample sources were downloaded by using the GEO query package [25] of R software (version 4.2.0) and served as discovery cohorts, including 2 miRNAs (GSE110993 and GSE117064), 1 mRNA (GSE58294), and 1 lncRNA (GSE198710) expression profile. In addition, the GSE95204 (miRNA), GSE102541 (lncRNA), and GSE16561 (mRNA) series were used as verification series. We carried out batch normalization to offset the deviations between two datasets using the sva [26] (v3.44.0) package in R. More detailed information about the six datasets is presented in Table 1. Additionally, a 396 human ferroptosis-related genes dataset was fetched from FerrDb (v2; http://www.zhounan.org/ferrdb) [27], including 207 drivers, 202 suppressors, and 3 markers (16 were overlapped genes among them) (See Additional file 1). As all the datasets were publicly accessible from the GEO database or FerrDb database, approval by the ethics committee of the First Affiliated Hospital of Guangxi Medical University was not required to conduct the current study. Thus, all data were freely available. The flowchart and data preprocessing are illustrated in Fig. 1.

**Table 1:** Detailed information on the studied gene expression profiles

Dataset	Platform	Control (n)	IS (n)	Samples	Application	Author	Country	Submission
GSE117064	GPL21263 3D-Gene Human miRNA V21_1.0.0	1612	173	Serum	Identification for DEmiRNAs	Sonoda T	Japan	2019
GSE110993	GPL15456 Illumina HiScanSQ (*Homo sapiens*)	20	20	Circulating blood	Identification for DEmiRNAs	Northoff BH	Germany	2018
GSE58294	GPL570 Affymetrix Human Genome U133 Plus 2.0 Array	23	69	Whole blood	Identification for DEmRNAs	Stamova B,	USA	2014
GSE198710	GPL21827 Agilent-079487 Arraystar Human LncRNA microarray V4	5	5	Whole blood	Identification for DElncRNAs	Jiang W	China	2022
GSE95204	GPL18058 Exiqon miRCURY LNA microRNA array, 7th generation	3	6	Circulating blood	Validation for key miRNAs	Deng Z	China	2017
GSE66724	GPL570 Affymetrix Human Genome U133 Plus 2.0 Array	8	8	Peripheral blood cells	Validation for key mRNAs	Allende M	Spain	2015
GSE16561	GPL22755 Agilent-076500 Human lncRNA+mRNA array	3	6	Whole blood	Validation for key lncRNA	Tian C	China	2017

*Abbreviations*: *IS* Ischemic stroke, *n* Number, *DEmiRNAs* Differentially expressed miRNAs, *DemRNAs* Differentially expressed mRNAs; differentially expressed lncRNA: DElncRNAs

### Differential expression analysis

We analyzed the expression matrixes of miRNA, lncRNA, and mRNA microarray. Differential expression analysis of the miRNA dataset of GSE110993 was provided in the GEO datasets. Other microarrays were reannotated using the platform annotation file. The probe annotation of this series was based on Entrez Gene ID. The data were quantile normalized, and the differential expressed miRNAs (DEmiRNAs), mRNAs (DEmRNAs), and lncRNAs (DElncRNAs) between the IS and control samples were identified using R’s limma package (v3.52.0) [19]. The *P* < 0.05, |log 2-fold change (FC)| > 0.5 were selected as cut-off criteria in identifying DEmRNAs and DEmiRNAs by previously reported methods [10, 21, 28–30], and |log 2-fold change (FC)| > 1 was selected to identify DElncRNA [31, 32]. Thereafter, volcano plots of DEmiRNAs, DElncRNAs, and DEmRNAs were generated using the ggplot2 package (v3.3.6) [33].

### Prediction of interactions of lncRNAs-miRNAs and miRNAs-mRNAs

First, using the Venn diagram online drawing tool (v2.1.0; http://bioinformatics.PSB.ugent.be/webtools/Venn), a Venn diagram was drawn to determine the DEmiRNAs shared between the two miRNA datasets (GSE110993 and GSE117064). Second, the online databases were utilized to pair lncRNA–miRNA and mRNA–miRNA, respectively. The target mRNAs of the DEmiRNAs were predicted using the miRWalk (v2.0; http://mirwalk.umm.uni-heidelberg.de/), which is a comprehensive database for predicting miRNA target genes and verifying miRNA binding sites [32]. The target lncRNAs were predicted by the ENCORI (Starbase v3.0; https://starbase.sysu.edu.cn/), which is a database that specifically records the interactions between miRNAs and lncRNAs [9, 31, 34, 35].

### Construction of ceRNA network

We took the DEmRNAs that overlapped with the target mRNA as the differentially expressed target mRNAs (DETmRNAs) and the DElncRNAs that overlapped with the target lncRNA as the differentially expressed target lncRNAs (DETlncRNAs). Finally, the DETlncRNAs–DEmiRNAs and DEmiRNAs–DETmRNAs interactors were subsequently linked to constructing the ceRNA network using the Cytoscape software (v3.8.0) [36], which is an open-source network visualization software platform mainly used for the analysis, research, and design of complex biological networks. The nodes in the network represent DETmRNAs, DEmiRNAs, and DETlncRNAs, and the edges represent their interaction. We used the cytoHubba plugin (v 0.1) [37] in the Cytoscape software to identify the top 10 hub miRNAs from the ceRNA network using the topology degree approach.

### Functional enrichment analysis

Gene ontology (GO) analysis and Kyoto Encyclopedia of Genes and Genomes (KEGG) pathway analysis were used to predict functional roles and other shared features of the above DETmRNAs based on the online tools of the Database for Annotation, Visualization, and Integrated Discovery (DAVID) Bioinformatics Resources(v 6.8; https://david.ncifcrf.gov/home.jsp) [38]. *P* < 0.05 was set as a significant filtering criterion in the enrichment analysis. The results of the enrichment analysis were visualized by the R package ggplot2 package (v3.3.6) [33].

### Construction of a protein-protein interaction (PPI) network

Given that proteins are the biomacromolecules that execute functions in our bodies, the online Search Tool for the Retrieval of Interacting Genes (STRING) database (v11.5; https:// string-db.org/) [39] was applied to critically assess and estimate the interaction between the DETmRNAs. Cytoscape [36] was used to construct and visualize this PPI network. We used the cytoHubba plugin to identify the top 10 hub mRNAs from the PPI network using the topology Degree and MCC approach [37, 40].

### Immune infiltration analyses

CIBERSORT [41] is an analytical tool of the classic deconvolution approach based on linear support vector regression. It can estimate the composition and abundance of immune cells infiltrating the cerebral infarction sample of mixed cells using mRNA expression data. We performed CIBERSORT (https://cibersortx.stanford.edu/) and parallel (v4.2.0), e1071 (v1.7–9), and preprocess Core (v1.58.0) packages in R for analysis. The relative percentages of 22 immune cell infiltration matrices and the immune infiltration distribution results in each sample of cerebral infarction were visualized using a bar plot. The ggplot2 package (v3.3.6) [33] in R was utilized to reflect the infiltrating difference between the IS and control samples via a violin diagram. *P* < 0.05 was accepted as the filtering cut-off value.

### Screening of key biomarkers

The diagnosis prediction of IS addressed a classification problem (i.e., whether a sample was identified as IS or control) in this study. We analyzed the classification and prediction efficiencies of the selected top 10 hub miRNAs ranked by the degree in the ceRNA network in the external validation cohort (GSE95204). The pROC package (v1.18.0) [42] in R was used to draw the ROC curve, with the sensitivity as the ordinate and 1-specificity as the abscissa. The area under the curve (AUC) served as the main evaluation performance. The higher the AUC value, the better the diagnostic power. We identified hub miRNAs with an AUC value higher than 0.7 as key miRNAs. The key mRNAs were found at the intersection between the top 15 hub mRNAs of the PPI and the ferroptosis-related genes dataset, which were also regulated by the key miRNAs in the ceRNA network. Furthermore, the key lncRNAs were identified by the intersection of the DETlncRNAs of the key miRNAs in the ceRNA network.

### Diagnostic performance of key mRNAs and lncRNAs

We verified the diagnostic performance of the key mRNAs in the discovery dataset (GSE58294) and validation series (GSE16561) and the key lncRNAs in the discovery dataset (GSE198710) and validation series (GSE102541), using the pROC package [42] to distinguish patients with IS from the controls. Next, the AUC values and 95% confidence interval were calculated to verify the reliability of the diagnostic curve.

### Cell culture

The SH-SY5Y cells are human neuroblastoma cells sharing similar properties with neurons in morphology, neurochemistry, and electrophysiology [43], which are the most commonly used tool in human in vitro ischemic research [44]. In this study, the SH-SY5Y cell line was obtained from the Saibaikang Biotechnology Co. (Shanghai, China) and was cultured in Eagle’s minimum essential medium and Ham’s F12 medium (MEM/F12 1:1), supplemented with 10% fetal bovine serum (Gibco, CA, USA) and 1% penicillin-streptomycin mixed solution (Gibco, CA, USA) at 37 °C in a 5% CO_2_ incubator. The medium was changed every other day, and the cells were subcultured about twice a week. In order to lyse the adherent cells, they were grown to 80% confluence and rinsed with phosphate buffer saline before trypsinization. Thereafter, the SH-SY5Y cells were seeded in 6-well plates for 24 h and randomly divided into the control and model groups.

An application of Hydrogen peroxide (H2O2) was used to mimic neural injury [44]. The H2O2 was freshly prepared before each experiment. The model groups were exposed to H2O2 concentrations corresponding to the half maximal inhibitory concentration (IC50) value (400 μ g/mL) for 6 h [43, 45]. The control groups were cultured in an equal volume of MEM/F12 1:1 medium without H2O2.

### Cell viability assessment by cell counting kit-8 assay

The cell viability was detected using a cell counting kit-8 (CCK-8, Meilunbio, China) for testing the toxicity of H_2_O_2_ to SH-SY5Y cells. The cells were seeded into 96-well plates and treated with different concentrations of H_2_O_2_ ranging from 0 to 5000 μM for 6 h. Then 10 μ l CCK-8 regent in 90 μ l medium was added to each well and incubated at 37 C for 1 h. The optical density was measured at 450 nm using a microplate reader (Synergy H1, BioTek, USA).

### Real-time polymerase chain reaction (qRT-PCR) analysis

The relative expression level of key miRNAs, lncRNAs, and mRNAs was verified using qRT-PCR. Total RNA was extracted using the NucleoZol RNA reagent (Macherey-Nagel, Düren, Germany) from the SH-SY5Y cells of the control and model groups according to the manufacturer’s instructions. The RNA was eluted in 10 μL DEPC-treated water, quantified by Thermo Scientific NanoDrop 2000 spectrophotometer (Thermo Scientific Nanodrop, USA), and stored at − 80 °C. On one hand, the PrimeScript RT reagent kit with a gDNA Eraser (Code No. RR047A, Takara, Japan) was used for reverse transcription (37 °C for 60 min, 85 °C for 5 min) of the mRNAs. On the other hand, the PrimeScript RT Master Mix (Code No. RR036Q/A/B, Takara, Japan) was used to synthesize cDNA (37 °C for 15 min, 85 °C for 5 s, and followed by storage at 4 °C) for the miRNA. We performed qRT-PCR using TB Green Premix Ex Taq II (Code No. RR820Q/A/B, Takara, Japan), according to the specifications provided by the manufacturer, on the Applied Biosystems 7500 fluorescence quantitative PCR instrument (Applied Biosystems, USA). The relative expression levels of the RNAs were calculated using the 2^-ΔΔCt^ method. GAPDH and RNU6B (U6) were set as the internal control. The primer sequences are listed in Additional file 2.

### Statistical analyses

The statistical analyses were processed using the R software (version 4.2.0) or SPSS 23.0 statistical software (IBM Corporation, Armonk, NY, USA). The independent samples Student’s *t-*test was used to estimate normally distributed variables, while the Mann-Whitney U test was used to compare non-normally distributed variables. The ROC curves were visualized, and the corresponding AUC values of the ROC curves were calculated using the pROC package in R. For each study, *P* < 0.05 was considered a significant difference.

## Results

### Differentially expressed lncRNA, miRNA, and mRNA

**Fig. 2:**
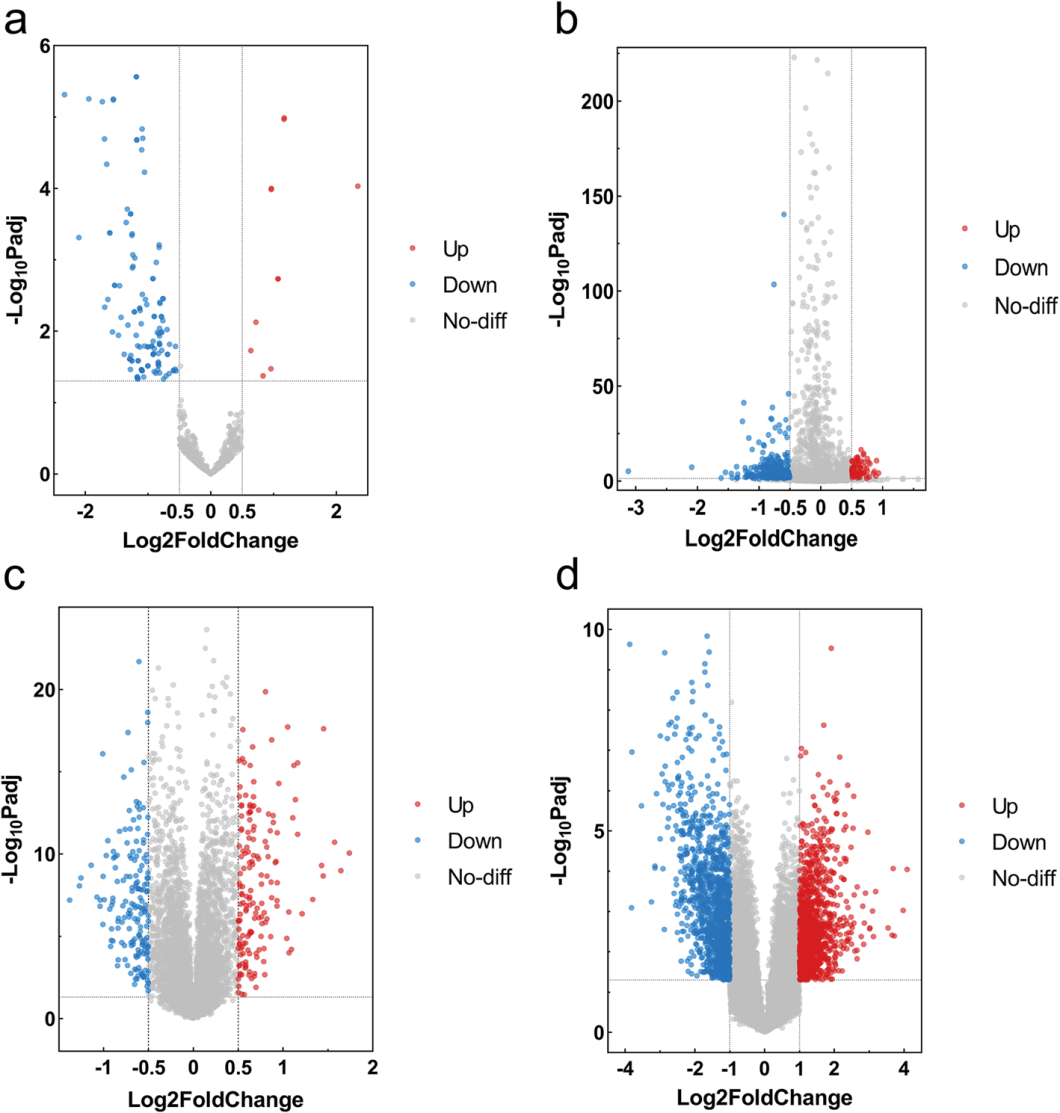
Volcano plots: red plot represented upregulation, and blue plot represented downregulation; **a** GSE110993; **b** GSE117064；**c** GSE58294；**d** GSE198710

The result of the differential expression analysis for GSE110993 was downloaded directly from GEO datasets. The expression matrixes of the other six datasets were obtained after pre-processing identically by background correction and normalization. We used R software to perform differential expression analysis on the gene expression matrix between stroke patients and healthy controls. In our study, a total of 140 DEmiRNAs in GSE110993, 430 DEmiRNAs in GSE117064, 334 DEmRNAs in GSE58294, and 2398 DElncRNAs in GSE198710 were identified. The results are shown in the volcano plots (Fig.2).

**Fig. 3:**
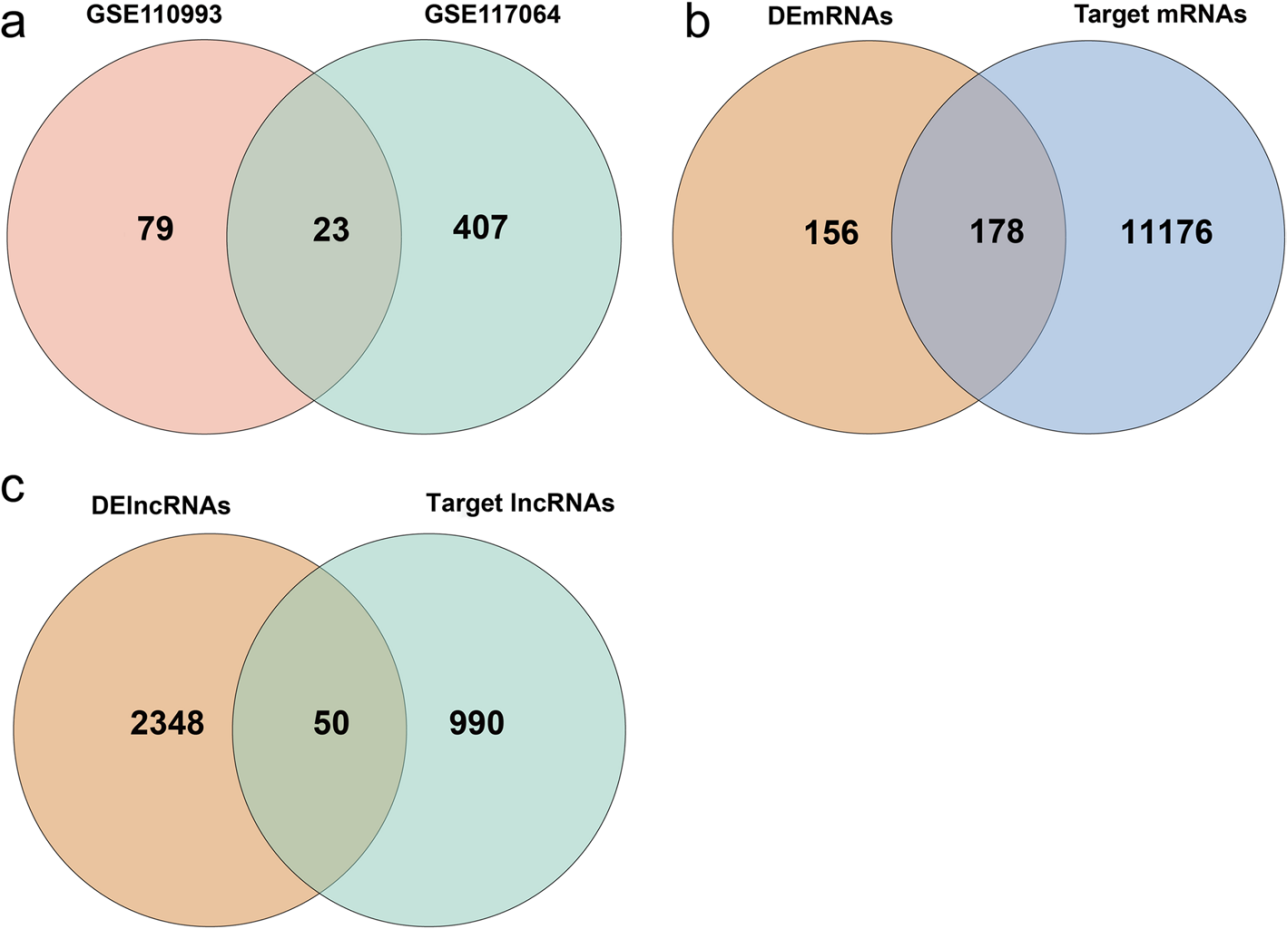
Venn diagrams: **a** Screen DEmiRNAs by the intersection of GSE110993 and GSE117064; **b** Screen DETmRNAs by the intersection of DEmRNAs and target mRNAs; **c** Screen DElncRNAs by intersection of DElncRNAs and target lncRNAs

Among the two miRNA datasets (GSE110993 and GSE117064), we found that 23 DEmiRNAs were changed consistently. These results were visualized by Venn diagrams (Fig. 3a). The StarBase displayed that 1040 target lncRNAs had binding sites with 17 miRNAs among the identified 23 DEmiRNAs. And the prediction of miRWalk databases demonstrated that 19 obtained miRNAs could bind to 11,354 target mRNAs among the aforesaid 23 DEmiRNAs. Based on the differential expression results and the prediction results, the intersection of the DEmRNAS and the target miRNAs was drawn (Fig. 3b) and identified as the DETmRNAs, as well as the intersection of the DElncRNAs and the target lncRNAs was drawn (Fig. 3c) and identified as the DETlncRNAs.

### Construction of ceRNA network

**Fig. 4:**
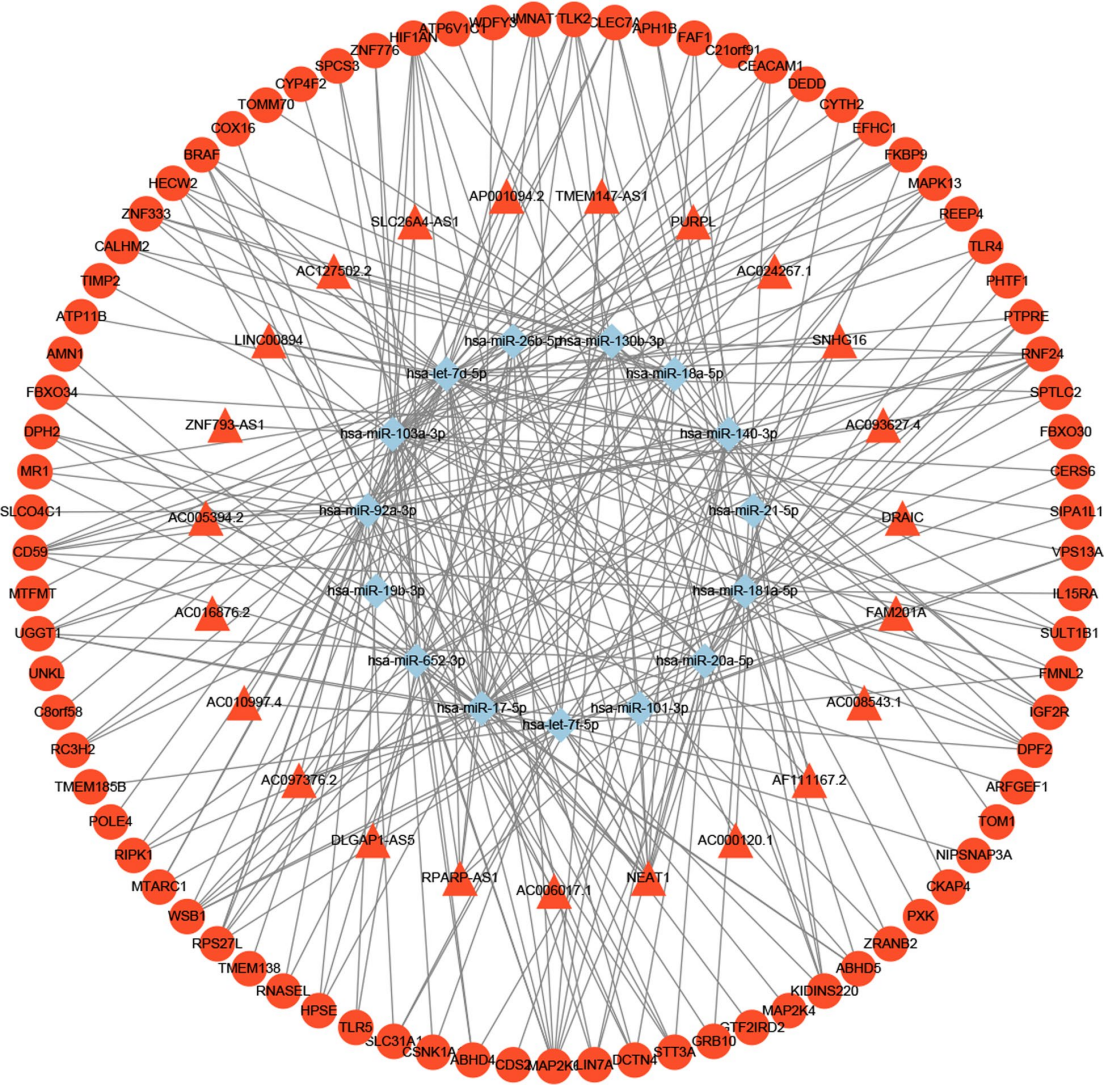
IncRNAs-miRNAs-mRNAs regulatory network. Every node symbolizes one gene, and each edge indicates the interaction between genes. The shape of triangle represents IncRNAs, diamond represents miRNAs, and regular hexagon represents mRNAs. The blue color genes symbolize downregulated genes, while the red color indicates upregulated genes

The obtained lncRNA-miRNA and mRNA-miRNA interactions were integrated to construct the lncRNA-miRNA-mRNA regulatory network. The ceRNA network of LncRNA-miRNA-mRNA interaction was constructed via 219 nodes consisting of 15 miRNAs, 44 lncRNAs, and 160 mRNAs, and 503 edges represented lncRNA-miRNA and miRNA-mRNA interactions. The detailed information was shown in Fig. 3 and Additional file 3. In the network, has-let-7d-5p (Degree = 64), has-miR-92a-3p (Degree = 60), has-miR-103a-3p (Degree = 55), has-miR-17-5p (Degree =51), has-let-7f-5p (Degree =38), has-miR-140-3p (Degree =37), has-miR-652-3p (Degree =37), has-miR-18a-5p (Degree =32), has-miR-20a-5p (Degree =28) and has-miR-130b-3p (degree = 27) were identified as the top 10 hub miRNAs (Table 2). The regulatory relationship between lncRNA, miRNA, and mRNA in the ceRNA network is shown in Fig.4.

**Table 2:** The top 10 hub RNAs in the ceRNAs network ranked by Degree

Number	Rank	Gene name	Node degree	Gene type
1	1	hsa-let-7d-5p	64	miRNA
2	2	hsa-miR-92a-3p	60	miRNA
3	3	hsa-miR-103a-3p	55	miRNA
4	4	hsa-miR-17-5p	51	miRNA
5	5	hsa-let-7f-5p	38	miRNA
6	6	hsa-miR-140-3p	37	miRNA
7	6	hsa-miR-652-3p	37	miRNA
8	8	hsa-miR-18a-5p	32	miRNA
9	9	hsa-miR-20a-5p	28	miRNA
10	10	hsa-miR-130b-3p	27	miRNA

### Functional enrichment analysis

**Fig. 5:**
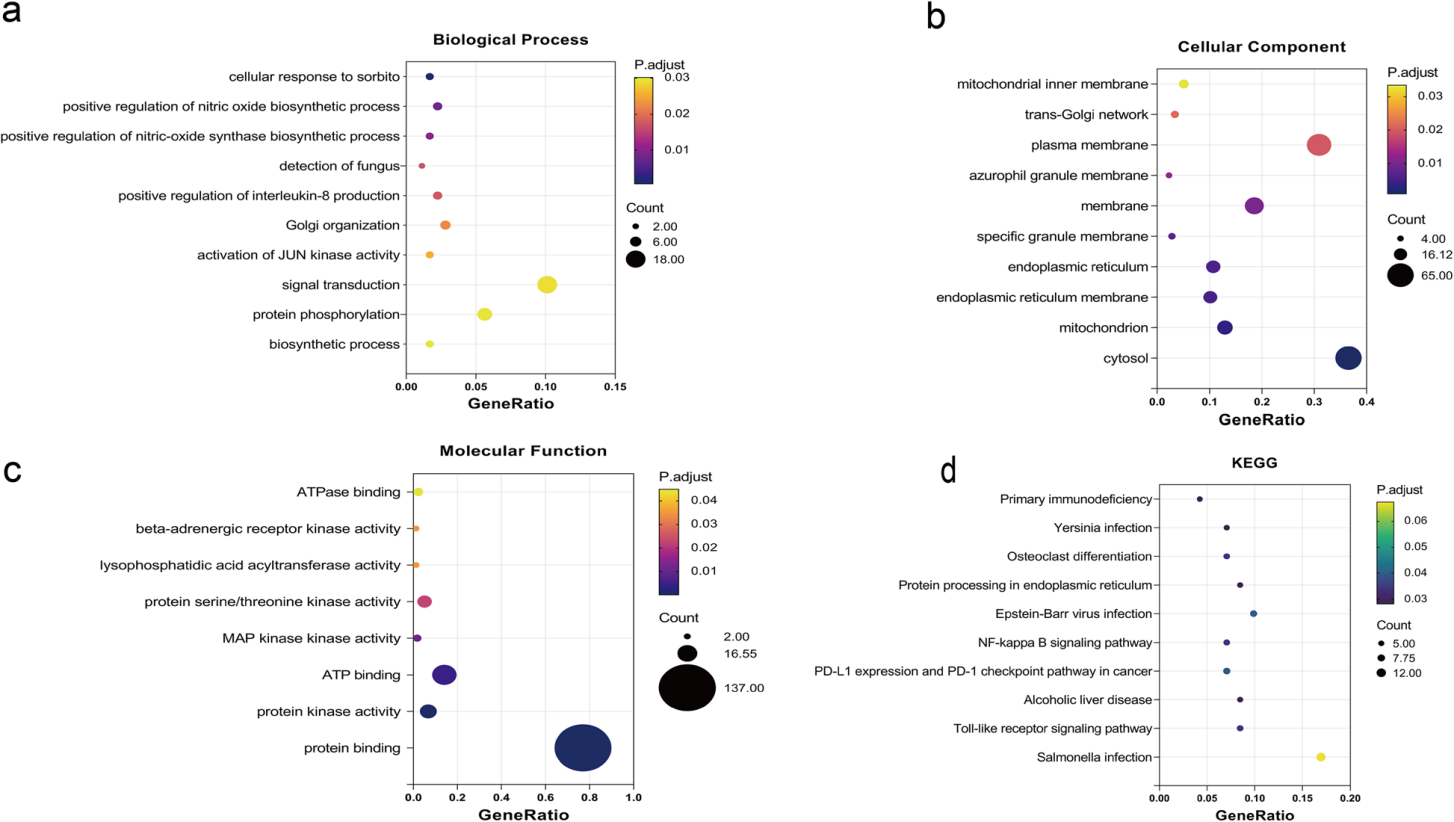
GO/KEGG function enrichment analysis: **a** biological process; **b** cellular component; **c** molecular function; **d** KEGG enrichment analysis (www.kegg.jp/kegg/kegg1.html)

GO and KEGG examinations on the 178 DETmRNAs were conducted. Both GO and KEGG enrichment analyses indicated that inflammatory genes may be crucial for IS development. We set adjusted *P*-value < 0.05 and ranked by *P*-value size, selecting the top 10 enrichment features for presentation. The biological process (BP) terms include cellular response to sorbitol, positive regulation of nitric oxide biosynthetic process, positive regulation of nitric-oxide synthase biosynthetic process, positive regulation of interleukin-8 production, and so on. The cellular component (CC) reveals the terms of cytosol, mitochondrion, endoplasmic reticulum membrane, endoplasmic reticulum, and so on. The molecular function (MF) terms are mainly associated with protein binding, protein kinase activity, ATP binding, MAP kinase activity, and so on. KEGG pathway analysis has shown that inflammatory-related functions, such as the Toll-like receptor signaling pathway and NF-kappa B signaling pathway, were significantly enriched. Detailed information on GO analysis and KEGG pathway analysis were shown in Fig. 5.

### PPI network of DETmRNAs

**Fig. 6:**
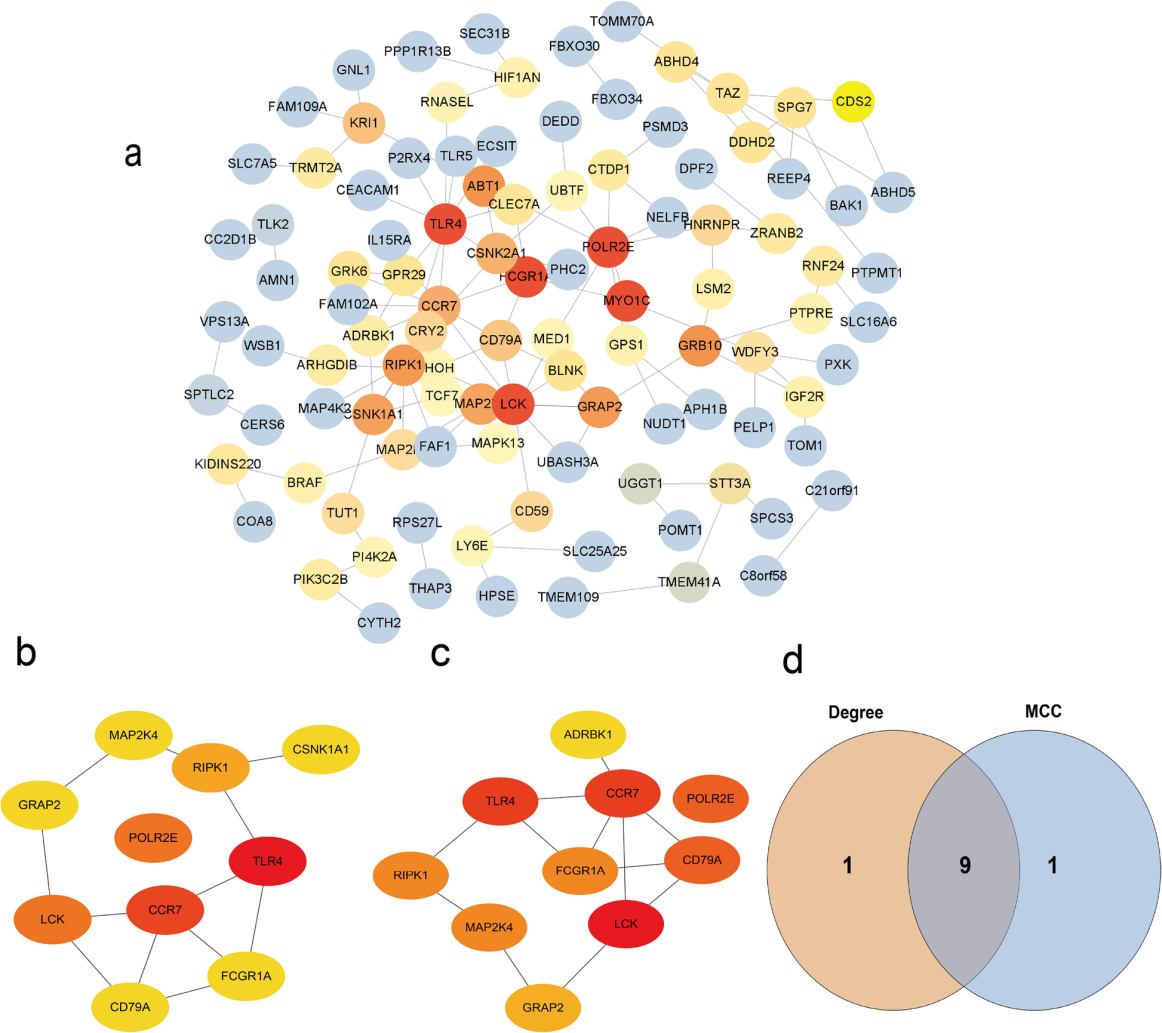
**a** a protein-protein interaction network; **b** Top 10 mRNAs ranked by Degree score; **c** Top 10 mRNAs ranked by MCC score; **d** Screen hub mRNAs by intersection of the top 10 in Degree and MCC

Using the STRING database, a total of 120 PPI interaction logs were predicted from the 178 DETmRNAs used to construct the PPI network (Fig.6a). The Degree and MCC topological feature were performed to calculate the top 10 ranking mRNAs respectively (Fig.6b, c, Table 3). Then, 9 mRNAs (TLR4, CCR7, LCK, POLR2E, RIPK1, MAP 2 K4, CD79A, GRAP2, FCGR1A) were identified as hub mRNAs by overlapping the top 10 of Degree and MCC (Fig. 6d). Of these mRNAs, 3 of them (TLR4, LCK, RIPK1) were linked to the NF-kappa B signaling pathway, and 3 of them (TLR4, RIPK1, MAP 2 K4,) were linked to the Toll-like receptor signaling pathway. These findings suggested that these hub mRNAs may be related to inflammation-related functions.

**Table 3:** The top 15 hub mRNAs in the PPI network ranked by Degree and MCC

	Degree			MCC		
Number	Rank	Gene name	Degree	Rank	Gene name	MCC
1	1	TLR4	10	1	LCK	13
2	2	CCR7	9	2	CCR7	12
3	3	LCK	8	2	TLR4	12
4	3	POLR2E	8	4	CD79A	8
5	5	RIPK1	6	4	POLR2E	8
6	6	MAP2K4	5	6	MAP2K4	7
7	6	CSNK1A1	5	6	FCGR1A	7
8	6	FCGR1A	5	6	RIPK1	7
9	6	GRAP2	5	9	GRAP2	6
10	6	CD79A	5	10	ADRBK1	5

### Immune infiltration landscapes

**Fig. 7:**
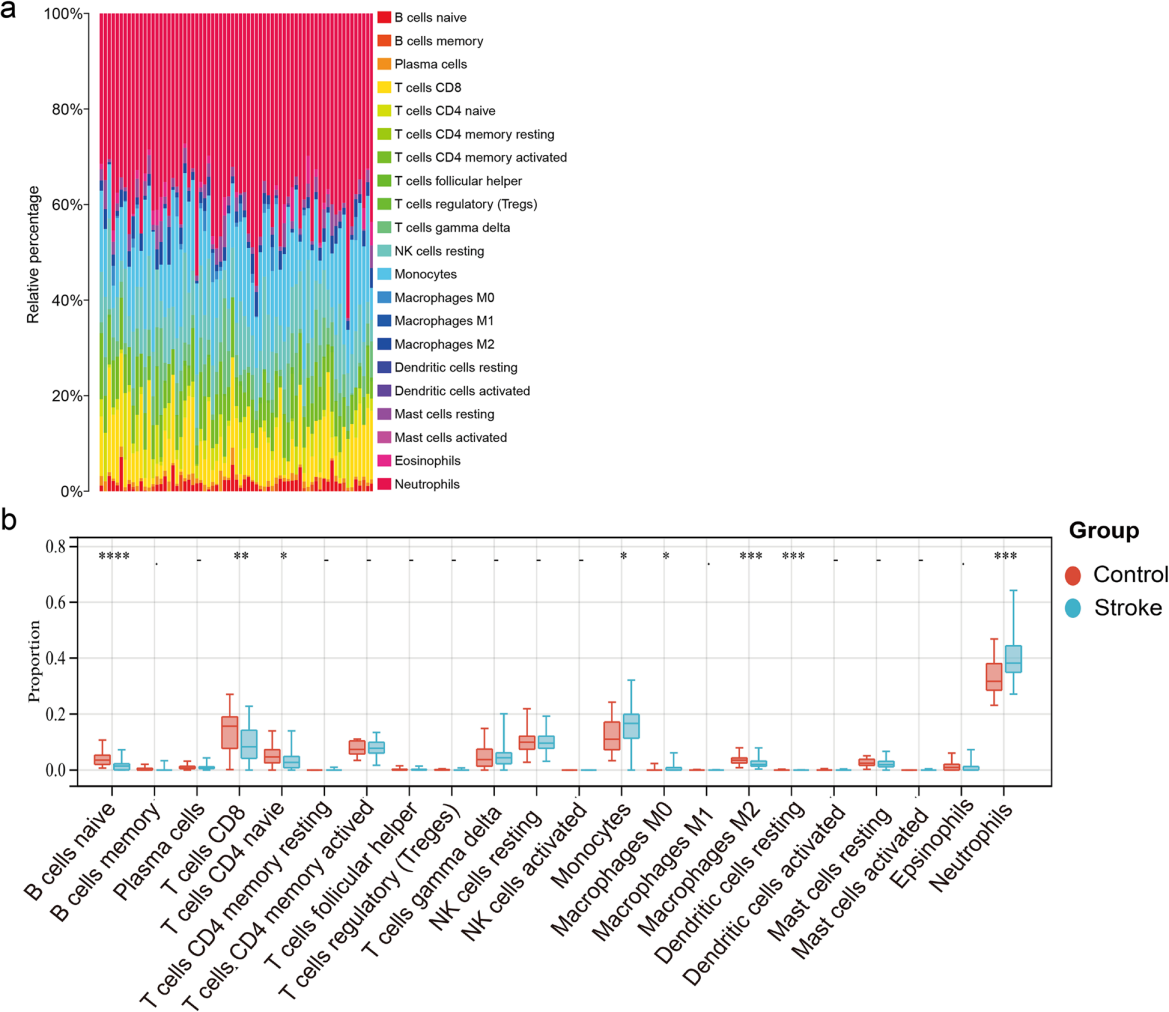
Evaluation and visualization of immune cell infiltration: **a** Immune cell infiltration map of cerebral infarction group; **b** Immune cell infiltration map between acute cerebral infarction group and control group. *P < 0.05, **P < 0.01, ***P < 0.001, ****P < 0.0001

We obtained the immune cell infiltration matrix and the results of immune infiltration distribution in IS samples. The bar plot clearly showed the contents of varied subpopulations in each individual (Fig. 7a). The violin diagram showed the infiltration of B cells naïve, T cells CD8, T cells CD4 naïve, monocytes, macrophages M0, macrophages M2, dendritic cells resting, and neutrophils in the cerebral infarction samples significant difference compared with the control group (Fig. 7b).

### Diagnostic performance of key miRNAs, mRNA, and lncRNA in IS

**Fig. 8:**
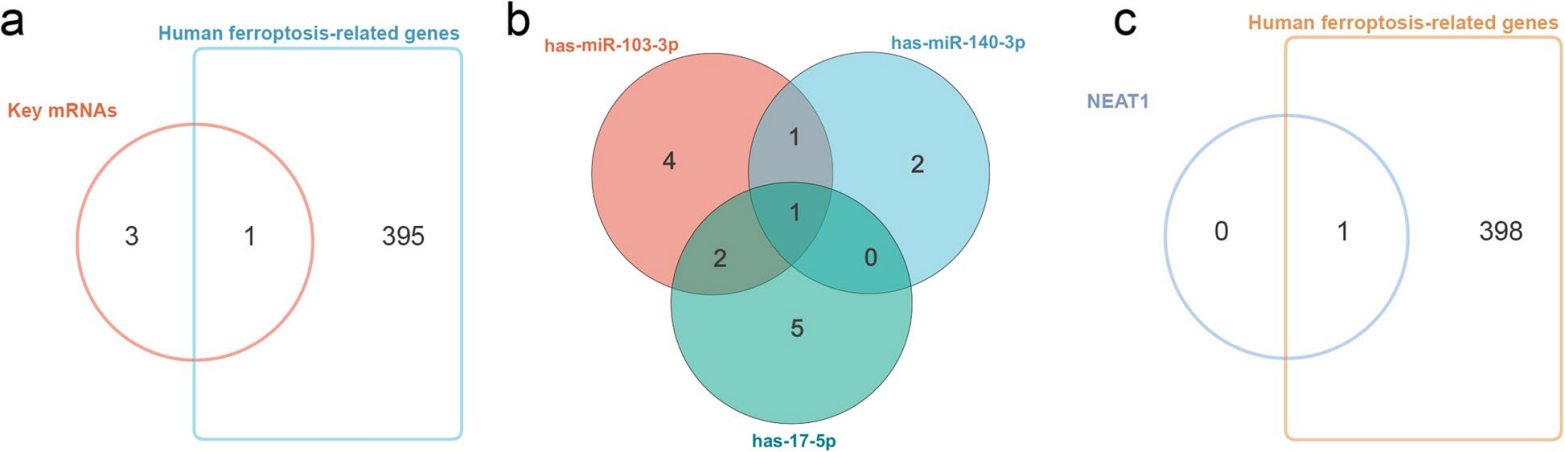
Screen for key mRNA and lncRNA: **a** Screen potential ferroptosis-related mRNA by the intersection of human ferroptosis-related genes and potential key mRNAs; **b** NEAT1 was identified by intersection of DETlncRNAs for the 3 key miRNAs; **c** NEAT1 happens to be contained in human ferroptosis-related genes

**Fig. 9:**
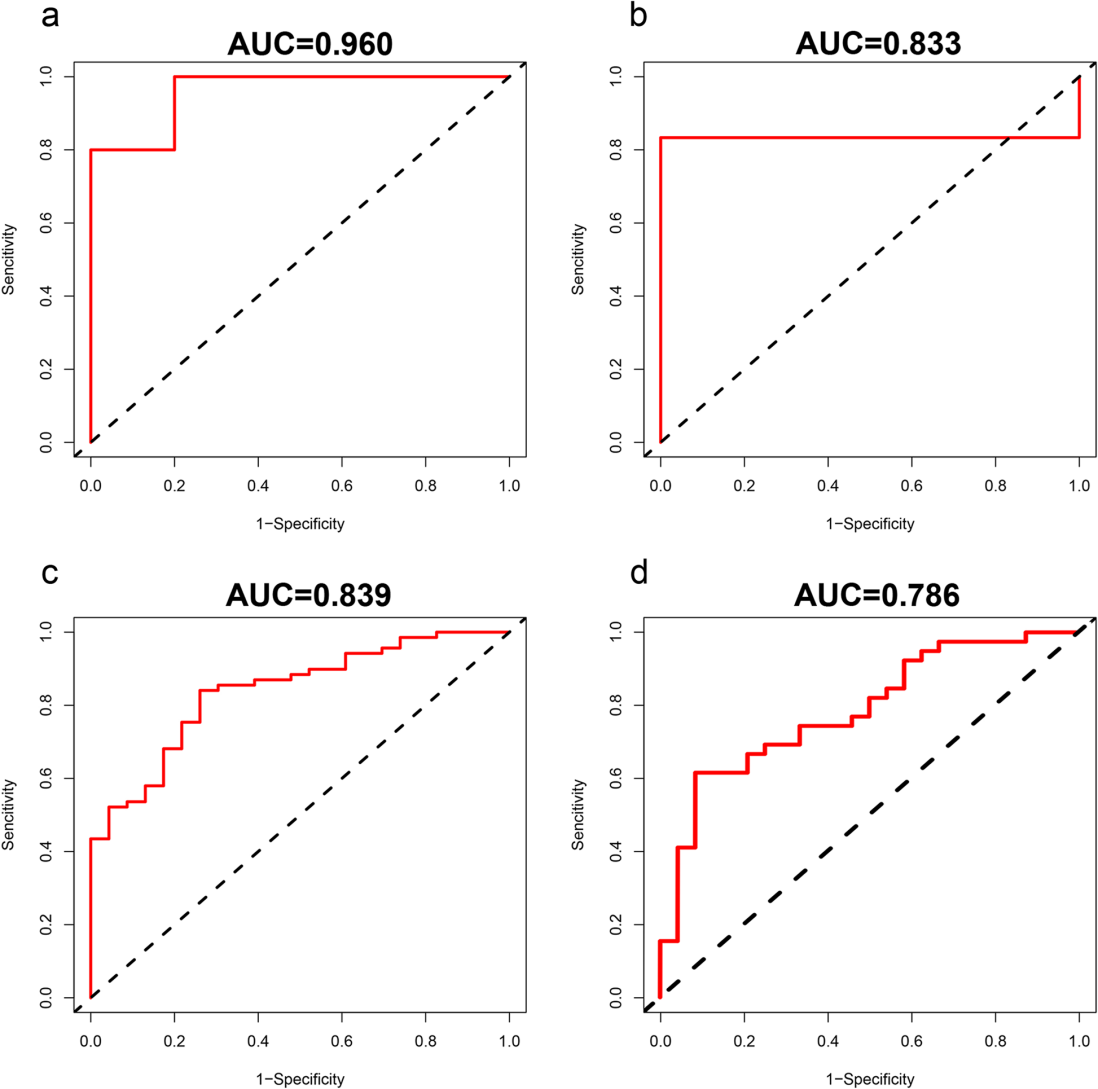
ROC curve of key lncRNA and mRNA: **a** NEAT1 in the discovery dataset (GSE198710);**b** NEAT1 in the validation set (GSE102541); **c** TLR4 in the discovery dataset (GSE58294) and **d** in the validation set (GSE16561)

The diagnostic performances of the top 10 hub miRNA to distinguish patients with IS and controls were appraised via ROC analysis in the validation cohort (GSE95204). We identify miR-103a-3p (AUC = 0.722, 95% CI = 0.260–1.000), miR-140-3p (AUC = 0.722, 95% CI = 0.260–1.000), and miR-17-5p (AUC = 0.833, 95% CI = 0.535–1.000) as key miRNAs (See Additional file 4). Then, the potential key mRNAs were obtained according to the PPI and ceRNA network, including TLR4, LCK, POLR2E and GRAP2 (see Additional File 3). After intersecting with human ferroptosis-related genes, we focused on one potential ferroptosis-related mRNA (TLR4) for further analysis (Fig. 8a). We screened the hub lncRNAs by the intersection of the differentially expressed target lncRNAs of the key miRNAs in the ceRNA network. Next, NEAT1 was identified as a key lncRNA by the intersection of the DETlncRNAs of the three key miRNAs (Fig. 8b), which happened to be contained in human ferroptosis-related genes (Fig. 8c). After that, ROC curve analysis was applied to evaluate the predictive performance of the key lncRNA. The AUC was 0.960 (95% CI = 0.843–1.000) for NEAT1 in the discovery dataset (GSE198710) and 0.833 (95% CI = 0.535–1.000) in the validation set (GSE102541) (Fig. 9a, b). Finally, ROC curve analysis was also applied to evaluate the potential diagnostic value of the one key mRNA (TLR4). The AUC of TLR4 was 0.839 in the discovery dataset (GSE58294) and 0.786 in the validation series (GSE16561) (Fig. 9c, d).

### H2O2 sensitivity of SH-SY5Y

**Fig. 10:**
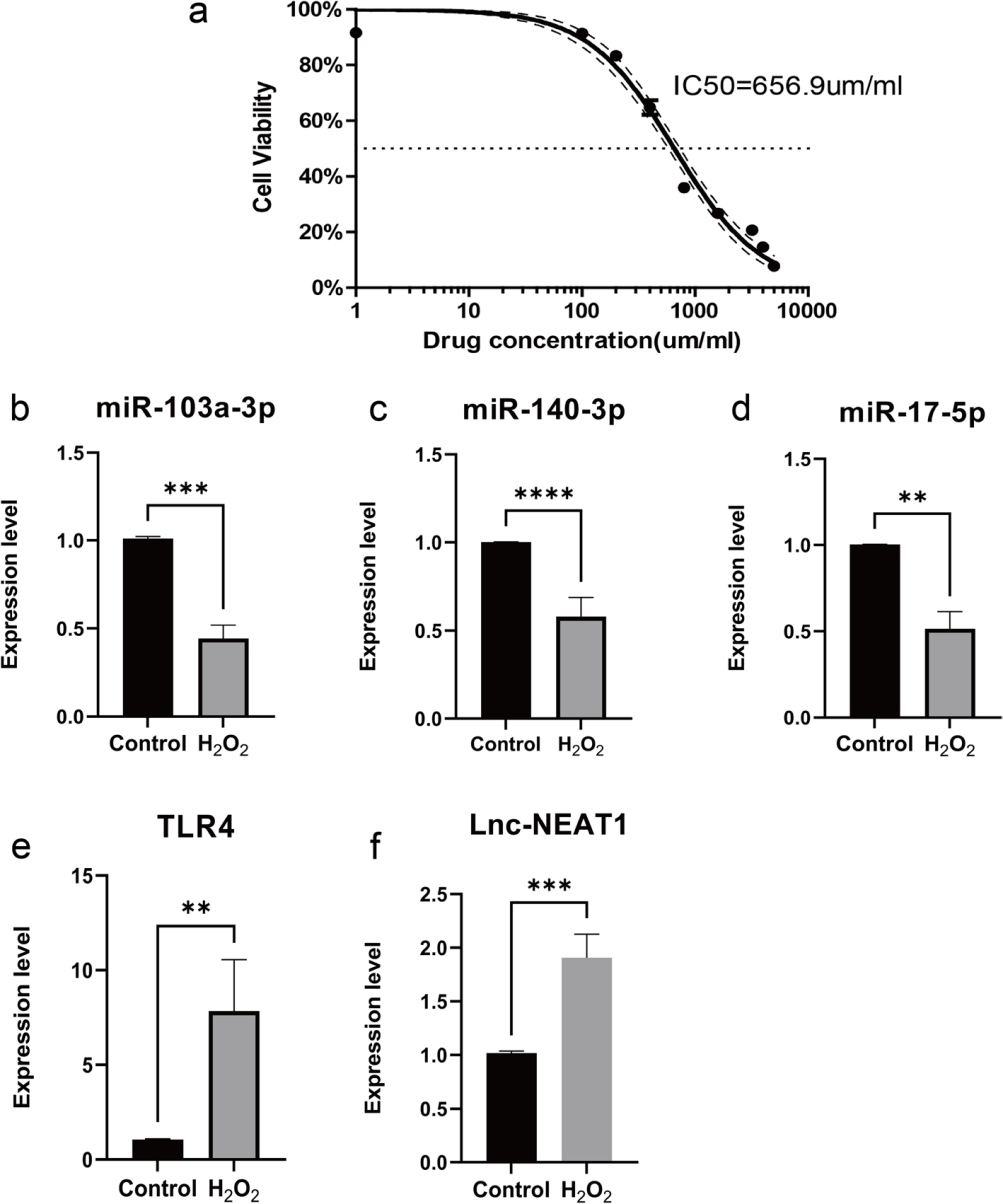
The relative expression of differentially expressed miRNA，mRNA, and lncRNA in SH-SY5Y cell: **a** Viability of SH-SY5Y cells after 6 h treatment with various concentrations of H2O2 (1 to 5000 μ M), estimated by CCK-8 assay; **b** miR-103a-3p; **c** miR-140-3p; **d** miR-17-5p; **e** TLR4; **f** NEAT1. The control group reflects the normal SH-SY5Y and the H2O2 group reflects the model, *P < 0.05, **P < 0.01, ***P < 0.001

The half-maximal inhibitory concentration (IC50), that is, the concentration of a drug that inhibits cell growth by 50% in different treatments [10]. In this study, the CCK-8 assay was used to test the toxicity of H2O2 to SH-SY5Y cells. As shown in Fig. 10a, the H2O2 showed a dose-dependent cytotoxicity against SH-SY5Y cells with IC50 values of 656.9 μ M.

### qRT-PCR verification of the identified biomarkers

The relative expression level of 3 the miRNAs (miR-103a-3p, miR-140-3p, miR-17-5p), 1 lncRNA (NEAT1), and 1 mRNA (TLR4) were verified using qRT-PCR. As predicted, the relative expression level of these biomarkers in the model groups was significantly different from the control groups (*P*-value< 0.05) (Fig. 10b-f).

## Discussion

IS remains a leading cause of permanent disability and causes a tremendous burden on human health and the economy [21]. Despite remarkable advances in the development of therapeutic and rehabilitation strategies, treatment efficacy remains unsatisfactory, and a considerable number of patients continue to suffer from long-term disability [11]. Several in vitro and in vivo studies have found that biomarkers involved in the inflammatory and ferroptosis processes may play crucial roles in the pathogenesis, prognosis, and treatment of stroke [14, 31]. Therefore, identifying inflammatory and ferroptosis-related biomarkers and developing effective targeted molecular therapies will greatly increase the opportunity for patients to receive effective post-stroke rehabilitation [21]. In our study, seven groups of RNA-sequencing results were combined to construct a ceRNA network through a comprehensive analysis, and five key RNAs were identified as potential biomarkers.

There is growing evidence that IS is commonly associated with the activation of an inflammatory response, which leads to increased expression of various pro-inflammatory cytokines and the subsequent amplification of neuronal cell death [8]. In our study, the functional annotation of 178 DETmRNAs revealed enrichment from GO and KEGG pathway analyses regarding the inflammatory response. Interestingly, there was also many evidence to support the enrichment results were associated with the mechanism of ferroptosis. Biological process analysis in GO annotations showed that DETmRNAs were mainly enriched in interleukin-8 (IL-8) production, a nitric oxide (NO) biosynthesis process, and a nitric oxide synthase (NOS) biosynthesis process associated with the inflammatory response. Several experimental studies have shown that IL-8 is a pro-inflammatory phase cytokine that acts as a chemoattractant for neutrophils [46]. Following IS, IL-8 levels increase, mobilize, and activate neutrophils, causing neutrophils to infiltrate the ischemic area and aggravating the local inflammatory response and brain tissue damage [8, 47]. In ovarian cancer, IL-8 level has been found to affect the metastatic process of ferroptosis-resistant cells [48]. In addition, reduced synthesis and reduced bioavailability of endothelial-derived NO were inextricably linked to the pathology of IS [49]. NO is constitutively synthesized by endothelial NOS and is a key gas transmitter for maintaining cerebrovascular homeostasis. NO maintains endothelial function by promoting vasodilation and preventing vascular smooth muscle cell proliferation, platelet aggregation, and leukocyte adhesion [50]. The biosynthesis process of NO has also been found to associate with brain tissue damage and neuronal ferroptosis in the pathology of cerebral ischemic/reperfusion [51]. The KEGG results showed that the DETmRNAs were mainly enriched in the Toll-like receptor signaling pathway and the NF kappa B signaling pathway. Together, these two signaling pathways are important mechanisms of inflammation. On one hand, the NF-kB signaling pathway can be activated by the previously mentioned pro-inflammatory cytokines to regulate inflammation, leading to increased brain damage [52]. Zhao et al. also showed that NF-κB inhibitors can significantly improve ischemia-induced neurological deficits [53]. On the other hand, Toll-like receptors (TLRs) are a family of innate immune system receptors that play an integral role in regulating systemic inflammatory responses. TLR signaling in immune cells, glia, and neurons have been shown to play a key role in the inflammatory cascade response following hypoxic-ischemic events, subsequently leading to the deleterious effects of neuroinflammation [54, 55]. Some researchers have found that inhibition of TLR signaling helps protect cortical neurons in the acute phase of ischemic injury. However, others have found that pharmacological pretreatment with TLR agonists before the induction of ischemic brain injury could also improve neuroprotection and reduce ischemic injury in various animal models [55]. Besides, these two pathways have been shown to be associated with ferroptosis in many studies [56–58]. For example, inflammatory responses after cardiac transplantation are initiated through ferroptotic cell death and TLR4/Trif-dependent signaling in graft endothelial cells [56]. Icariside II preconditioning evokes robust neuroprotection against ischemic stroke by targeting Nrf2 and the OXPHOS/NF-κB/ferroptosis pathway [58]. The above findings suggest that the therapeutic interventions targeting the ferroptosis, inflammatory response and modulation of neuronal signaling pathways may offer new opportunities for the future of IS treatment.

Pro-inflammatory signals from immune mediators rapidly activate resident cells and affect the infiltration of a wide range of inflammatory cells (neutrophils, monocytes/macrophages, different subtypes of T cells, among others) in the post-stroke ischemic area, which can exacerbate post-stroke brain injury and promote cell death [59]. Neutrophils are among the first immune cells to infiltrate the ischemic tissues after IS. They are attracted to ischemic areas by chemokines through concentration gradients and cause secondary injury through the release of pro-inflammatory factors, reactive oxygen species, proteases, and matrix metalloproteinases [60]. The functions of microglia and monocytes/macrophages vary according to the states of polarization. Several studies have shown that microglia and monocytes/macrophages may play a deleterious role in the acute phase of IS by accelerating inflammation. However, they have also been shown to play a protective role in the subacute and chronic phases, including neurogenesis, axonal growth, synaptogenesis, angiogenesis, and myelin regeneration [61]. Unlike neutrophils and monocytes, lymphocyte migration takes place during the chronic phase of IS [62]. Many studies have found that the frequency of lymphocytes (B cells and T cells) is significantly reduced in the acute phase but gradually recovers over time, which may be due to many causes, including activation of the sympathetic nervous system, dysregulation of the hypothalamic-pituitary-adrenal axis, and so on [63]. Dendritic cells (DC) play a crucial role in innate and adaptive immunity. Under physiological conditions, DCs are rarely present in the brain parenchyma, but they increase as the neuroinflammatory response increases [64]. The results of our immune infiltration analysis are consistent with those of previous studies in terms of more infiltration of monocytes, macrophages, DCs, and neutrophils and less infiltration of the number of B cells and T cells in the IS samples compared to the control.

Ferroptosis is a recently identified type of iron-dependent cell death [65]. A plethora of data has indicated that ferroptosis as a nexus linking inflammation plays an inevitable and important role in the pathogenesis of cardiovascular diseases such as stroke [11, 65]. Furthermore, the inflammatory responses that occur in central nervous system (CNS) trauma could induce the accumulation of lipid peroxidation as well as iron metabolism disorder to predispose neurons to undergo ferroptosis. In turn, the ferroptosis neurons themselves are also able to release damage-associated molecular patterns, further exacerbating the sterile inflammation response to aggravate the CNS damage in a vicious circle [66, 67]. On the one hand, our results identified three core miRNAs (miR-103a-3p, miR-17-5p, and miR-140-3p), one lncRNA (NEAT1), and one mRNA (TLR4) as the promising diagnostic markers in this study. The role of miR-103a-3p, miR-17-5p, and miR-140-3p has been widely explored in inflammatory responses for IS pathogenesis [19, 68, 69]. In addition, miR-17-5p has also been reported to associate with ferroptosis in the disease of brain metastasis in lung adenocarcinoma [70]. Nevertheless, few works have been dedicated to the study of these core miRNAs in ferroptosis for ischemic stroke. On the other hand, a potential regulatory axis for TLR4 has also been identified through a comprehensive analysis showing that NEAT1 could act as a sponge of miR-17-5P, competing to bind to up-regulated TLR4. Previous studies have found that NEAT1 is associated with the development, progression, and prognosis of IS [61, 71]. NEAT1 has been indicated as facilitating OGD/R injury and neuroinflammation damage of microglial cells as well as the excessive apoptosis of epithelial cells [71, 72]. NEAT1 has also been demonstrated to promote ferroptosis by modulating the miR-362-3p/MIOX axis as a ceRNA [73]. TLR4 is an innate immune protein widely expressed in nerve cells and plays a critical role in initiating neuroinflammatory responses and mediating neuroimmune responses [74]. Zhu et al. showed that TLR4 can be inappropriately activated by endogenous ligands released from injured tissue and dying cells following ischemic stroke. The inhibition of TLR4 suppressed the expression of ferroptosis-related proteins, decreased the activation of neuronal ferroptosis, and attenuated neuroinflammation response, ultimately increasing neuronal cell viability [75]. Therefore, further research on the mechanisms of ferroptosis as well as the link between ferroptosis and neuroinflammation will help provide new treatment targets for IS [66].

Our study does have several limitations. First, the sample size of each dataset was small, and therefore, additional high-throughput sequencing experiments with larger samples are needed to confirm the findings. Second, this study contained datasets from different testing platforms, which may have biased our results from those generated using data from the same platform. Third, this study only used ROC analysis and qRT-PCR to demonstrate the diagnostic and prognostic values; therefore, future mechanistic research should involve more experiments. Finally, the ceRNA mechanism should be validated by dual luciferase reporter gene assays and in vitro and in vivo knockdown or overexpression.

## Conclusion

In conclusion, this study constructed an IS-related ceRNA network and identified several signaling pathways associated with IS inflammation and biomarkers related to inflammation and ferroptosis, which are consistent with current pathological knowledge of the disease. We believe that this study provides a new understanding of the molecular mechanisms of IS pathogenesis and that the key RNAs identified herein may become new therapeutic targets for IS rehabilitation strategies.

## Supplementary Information

**Additional file 1.** The 396 human ferroptosis-related genes.

**Additional file 2.** Primer sequence for qRT-PCR.

**Additional file 3.** Interactions of lncRNA-miRNA and miRNA-mRNA.

**Additional file 4.** ROC curve of key miRNAs in the validation set:(a) has-miR-140-3p; (b) has-miR-103a-3p; (c) has-miR-17-5p; (d) has-miR-18a-5p; (e) has-let-5d-5p; (f) has-miR-7f-5p; (g) has-miR-652-3p; (h) has-miR-92a-3p.

## Data Availability

The datasets that support the findings of the current study are available in the GEO database (https://www.ncbi.nlm.nih.gov/geo/) and FerrDb database (http://www.zhounan.org/ferrdb). And they are also available from the corresponding author, upon reasonable request.

## Abbreviations

IS: Ischemic stroke
ncRNA: Non-coding RNA
miRNA: MicroRNA
mRNA: Messenger RNA
lncRNAs: Long non-coding RNAs
ceRNA: Competing endogenous RNAs
OGD/R: Oxygen and glucose deprivation/reoxygenation
GEO: Gene expression omnibus
ROC: Receiver operating characteristic
DEmiRNA: Differential expressed miRNA
DEmRNA: Differential expressed mRNA
DElncRNA: Differential expressed lncRNA
DETmRNA: Differentially expressed target mRNA
DETlncRNA: Differentially expressed target lncRNA
GO: Gene Ontology
DAVID: Database for Annotation, Visualization and Integrated Discovery
STRING: Search Tool for the Retrieval of Interacting Genes
PPI: Protein-Protein Interaction
AUC: Area under the curve
qRT-PCR: Real-Time Polymerase Chain Reaction
CCK-8: Cell counting kit-8
U6: RNU6B
BP: Biological process
CC: Cellular component
MF: Molecular function
IL-8: Interleukin-8
NO: Nitric oxide
NOS: Nitric oxide synthase
TLR: Toll-like receptor
DC: Dendritic cell
IC 50: Half maximal inhibitory concentration
CNS: Central nervous system
