# Supplementary material for: Competing endogenous RNA network analysis of the molecular mechanisms of ischemic stroke
Source: BMC Genomics. 2023 Feb 8;24:67. doi: 10.1186/s12864-023-09163-1 (PMC9906963; doi:10.1186/s12864-023-09163-1)
Supplement: Supplementary file 1 — Additional file 1. The 396 human ferroptosis-related genes. [file 12864_2023_9163_MOESM1_ESM.docx]

**The 396 human ferroptosis-related genes**

| **Data set** | **Gene** |
| --- | --- |
| **Marker** | PTGS2，CHAC1, FTH1 |
| **Suppressor** | SLC7A11, GPX4, AKR1C1, AKR1C2, AKR1C3, RB1, HSPB1, HSF1, NFE2L2, SQSTM1, NQO1, HMOX1, FTH1, MUC1, MT1G, SLC40A1, CISD1, HSPA5, ATF4, TP53, HELLS, SCD, FADS2, SRC, STAT3, PML, NFS1, TP63, CDKN1A, MIR137, VDAC2, FH, CISD2, MIR9-1, MIR9-2, MIR9-3, CBS, ISCU, ACSL3, OTUB1, CD44, LINC00336, BRD4, PRDX6, MIR17, SESN2, NF2, ARNTL, HIF1A, JUN, CA9, TMBIM4, PLIN2, AIFM2, LAMP2, ZFP36, PROM2, CHMP5, CHMP6, CAV1, GCH1, SIRT3, PIR, GCLC, HCAR1, SLC16A1, RRM2, NR4A1, PIK3CA, RPTOR, SREBF1, SREBF2, FZD7, P4HB, BCAT2, PLA2G6, MIR424, PARK7, FXN, SUV39H1, ATF2, STK11, FNDC5, CircIL4R, CDH1, MIR214, NEDD4L, TF, FTMT, BRD2, BRD3, BRDT, DECR1, GLRX5, NCOA3, NR5A2, MTOR, PANX2, RHEBP1, TFAP2A, CP, ARF6, GDF15, ABHD12, TFAM, KDM3B, RNF113A , AHCY, circ-TTBK2, MIR522, IDH2, PPARA, SIAH2, PRKAA2, NEDD4, PRDX1, AR, MTF1, COPZ1, NUPR1, USP35, NEAT1, PARP1,PARP2, PARP3, PARP4, PARP6, PARP8, PARP9, PARP10, PARP11, PARP12, PARP14, PARP15, PARP16, PDSS2, OIP5-AS1, MIR190A, CREB1, CREB3, CREB5, GOT1, MIR130B, BEX1, FABP4, AKT1S1, MLST8, SIRT1, TYRO3, SIRT6, TMSB4X, TMSB4Y, KIF20A, ECH1, circRHOT1, ETV4, MEG8, VCP, circ_0007142, RBMS1, KDM4A, MGST1, circKIF4A, miR-7-5p, circ_0067934, MPC1, CAMKK2, SOX2, SRSF9, MIR4443, MIR27A, MIR670, MEF2C, EZH2, PEDS1, CDC25A, G6PD, LCN2, TRIB2, DHODH, MIR545, PDK4, CircPVT1, circDTL, IL6, PTPN18, FTL, ABCC5, CISD3, FURIN, circRHBG, GALNT14, KLHDC3, LINC01833, circGFRA1, GSTM1, circ0097009, TMEM161B-DT, circEPSTI1, MIR18A, RARRES2, USP11 |
| **Driver** | RPL8, IREB2, ATP5MC3, CS, EMC2, ACSF2, NOX1, CYBB, NOX3,NOX4, NOX5, DUOX1, DUOX2, G6PD, PGD, VDAC2, TP53, ACSL4, LPCAT3, NRAS, KRAS, HRAS, CARS1, KEAP1, HMOX1, ATG5, ATG7, NCOA4, TF, ALOX5, ALOX12, ALOX12B, ALOX15, ALOX15B, ALOXE3, PHKG2, SAT1, EGFR, MAPK3, MAPK1, ZEB1, DPP4, CDKN2A, PEBP1, SOCS1, CDO1, MYB, SLC1A5, CHAC1, LINC00472, GOT1, BECN1, PRKAA2, PRKAA1, ELAVL1, BAP1, ABCC1, MIR6852, ACVR1B, TGFBR1, IFNG, ANO6, HMGB1, TNFAIP3, ATF3, ATM, YY1AP1, EGLN2, MIOX, TAFAZZIN, MTDH, IDH1, FBXW7, PANX1, DNAJB6, LONP1, CD82, IL1B, POR, CYB5R1, ELOVL5, FADS1, PTEN, IL6, miR-182-5p, miR-378a-3p, CTSB, ATF4, LINC00618, MT1DP, PEX10, AGPAT3, PEX12, CHP1, GPAT4, BRPF1, OSBPL9, INTS2, MMD, CYP4F8, MLLT1, TTPA, GRIA3, EPT1, POM121L12, LIG3, AEBP2, AGPS, CDCA3, PEX2, PEX6, TIMM9, DCAF7, LCE2C, FAR1, PHF21A, SMAD7, LYRM1, AMN, PEX3, MTCH1, SIRT1, ACADSB, PVT1, hsa_circ_0008367, GSK3B, MAPK8, BRD7, SLC25A28, SLC11A2, ZFAS1, SLC38A1, TSC1, TGFB1, SNCA, SIRT3, TFRC, CGAS, STING1, HDDC3, MIR761, MDM2, MDM4, MIR214, DLD, WWTR1, PRKCA, SMPD1,MYCN, IFNA1, IFNA2, IFNA4, IFNA5, IFNA6, IFNA7, IFNA8, IFNA10, IFNA13, IFNA14, IFNA16, IFNA17, FNA21, SMG9, TLR4, PAQR3, MICU1, TOR2A, MIR375, CircKDM4C, MIR324, QSOX1,CLTRN, KLF2, MIR5096, H19, YTHDC2, DDR2, SLC39A7, TRIM46, ACSL1, KDM5A, CYGB, GSTZ1, ACO1, GJA1, SLC7A11, CIRBP, circPSEN1, YAP1, TRIM26, NDRG1, MIR302A, ASMTL-AS1, FADS2, PIEZO1, LIFR, PTPN6, ADAM23, ARHGEF26-AS1, CPEB1, MIR15A, KDM6B, METTL14, MIB1, KDM5C, CCDC6, MIR539 |


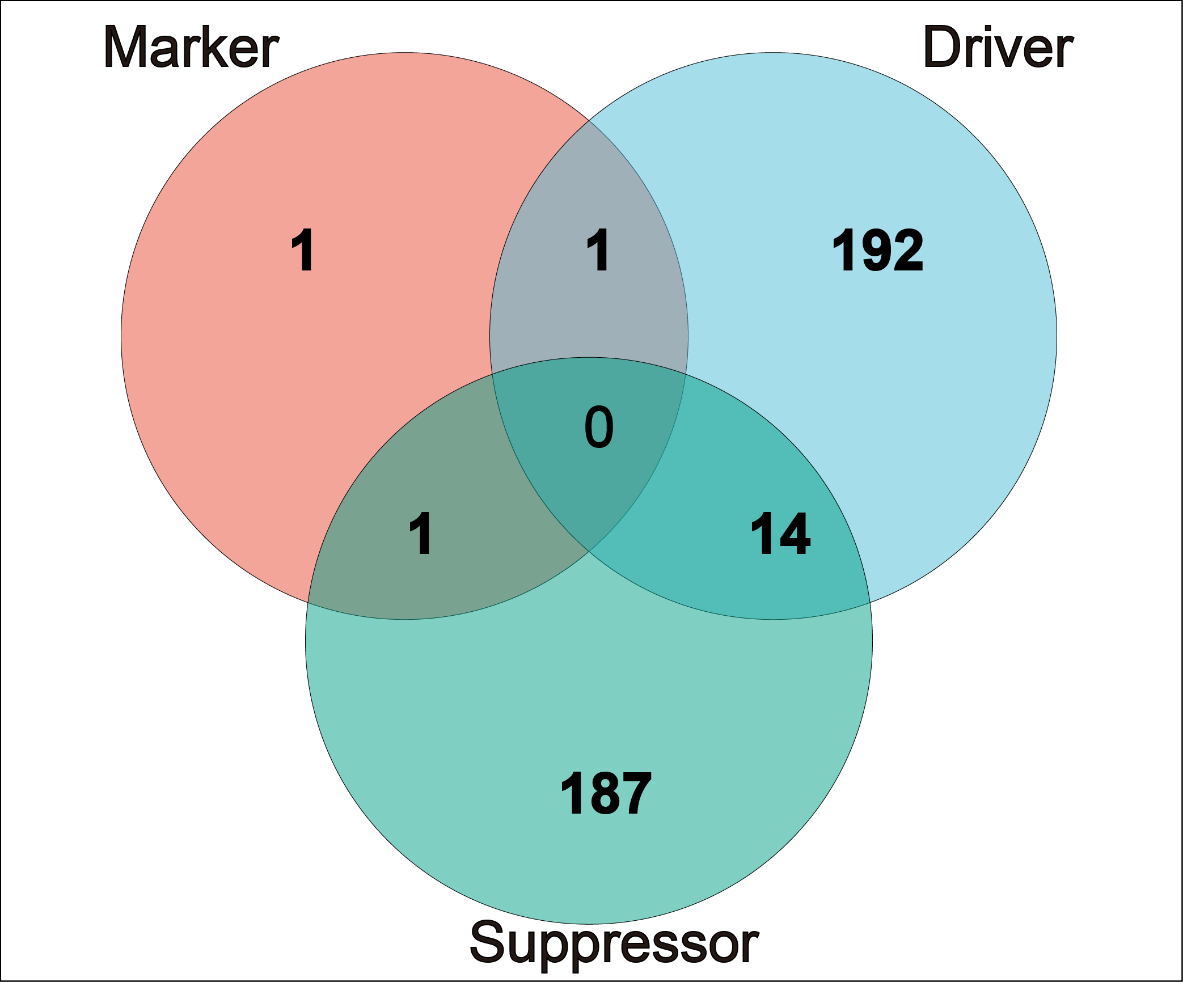


**396 human ferroptosis-related genes dataset: 207 drivers, 202 suppressors, 3 markers and 16 were overlapped genes among them.**
