# Supplementary material for: Competing endogenous RNA network analysis of the molecular mechanisms of ischemic stroke
Source: BMC Genomics. 2023 Feb 8;24:67. doi: 10.1186/s12864-023-09163-1 (PMC9906963; doi:10.1186/s12864-023-09163-1)
Supplement: Supplementary file 2 — Additional file 2. Primer sequence for qRT-PCR. [file 12864_2023_9163_MOESM2_ESM.docx]

**Primer sequence for qRT-PCR**

|  | Forward (5’-3’) | Reverse (3’-5’) |
| --- | --- | --- |
| miR-17-5p | GGCAAAGTGCTTACAGTGCAGGTAG | mRQ 3’primer (Takara, Japan) |
| miR-103-3p | AGCAGCATTGTACAGGGCTATGA | mRQ 3’primer (Takara, Japan) |
| miR-140-3p | GTACCACAGGGTAGAACCACG | mRQ 3’primer (Takara, Japan） |
| TLR4 | ACCATCATTGGTGTGTCGGT | GGCCACACCGGGAATAAAGT |
| NEAT1 | CTCTAGGTTTGGCGCTAAACTCTT | CCACCATTACCAACAATAACCGACT |
| GAPDH | CAGGAGGCATTGCTGATGAT | GAAGGCTGGGGCTCATTT |
| U6 | GGAACGATACAGAGAAGATTAGC | TGGAACGCTTCACGAATTTGCG |
