# Supplementary material for: Competing endogenous RNA network analysis of the molecular mechanisms of ischemic stroke
Source: BMC Genomics. 2023 Feb 8;24:67. doi: 10.1186/s12864-023-09163-1 (PMC9906963; doi:10.1186/s12864-023-09163-1)
Supplement: Supplementary file 3 — Additional file 3. Interactions of lncRNA-miRNA and miRNA-mRNA. [file 12864_2023_9163_MOESM3_ESM.docx]

**Interactions of lncRNA-miRNA and miRNA-mRNA**

| **Number** | **DEmiRNA** | **lncRNA-**  **miRNA pairs** | **miRNA-mRNA pairs** | **DETlncRNA** | **DETmRNA** | **Overlap between DETmRNA**  **and hub mRNAs of PPI** |
| --- | --- | --- | --- | --- | --- | --- |
| 1 | hsa-let-7d-5p | 13 | 52 | AC239868.1, MUC20-OT1, HEIH, AC010997.4, NUTM2A-AS1, RPARP-AS1, NEAT1, AF111167.2, DRAIC, AC009022.1, SNHG16, TMEM147-AS1, LINC00894 | FAM102A, BRAF, CD59, MTARC1, MAP2K6, DPF2, SPTLC2, MAPK13, CLEC7A, HECW2, PEDS1, PPIL2, ZNF710, MTFMT, POLR2E, WSB1, LIN7A, IGF2R, PELP1, RNF24, CSNK2A1, FKBP9, TLK2, CRY2, GRK2, TAFAZZIN, BAK1, PEX26, RHOH, ZNF333, UGGT1, TBRG4, ATXN3, DDHD2, MAP4K2, NMT2, SLC25A25, PUS1, RPS27L, DCTN4, FMNL2, USP24, PTPRE, SUGP2, EFHC1, SPG7, TCF7, NMNAT1, CALHM2, THEM4, UNKL, TMEM138 | POLR2E |
| 2 | hsa-let-7f-5p | 13 | 25 | AC239868.1, MUC20-OT1, HEIH, AC010997.4, NUTM2A-AS1, RPARP-AS1, NEAT1, AF111167.2, DRAIC, AC009022.1, SNHG16, TMEM147-AS1, LINC00894 | MAP2K4, CCR7, PPIL2, ATXN3, VPS13A, FMNL2, THEM4,WSB1, THAP3, GOLGA8Am CLEC7A, USP24, AHCYL2, RPS27L, TMEM104, HIF1AN, PI4K2A, UGGT1, CD79A, NMNAT1, TMEM185Bm SLC31A1, CSNK2A1, PIK3C2B, KIDINS220 | MAP2K4, CCR7, CD79A |
| 3 | hsa-miR-101-3p | 3 | 7 | FAM201A, NEAT1, AL163932.1 | PHTF1, HIF1AN, MYO1C, TLK2, PEX26, SCRN1, VPS13A | - |
| 4 | hsa-miR-103a-3p | 8 | 47 | NUTM2A-AS1, RPARP-AS1, NEAT1, AL049775.1, AC024267.1, AP001094.2, TMEM147-AS1, ZNF793-AS1 | TIMP2,CD59, MAP2K6, LCK, DPF2, CSNK1A1, ABHD5, SIPA1L1, TMEM109, GNL1, IGF2R, CYTH2,HIF1AN, ZNF776, ITM2C, FKBP9, NELFB, CRY2, C8orf58, RNASEL, STT3A, SULT1B1, TLR4, DEDD, PPP1R13B, PDAP1, ZNF333, HPCAL1, SLC41A1, SPCS3, DDHD2, VPS13A, MED1, RPS27L, POLE4, ABHD4, LY6E, ZRANB2,  CERS6, EFHC1, TCF7, TRMT2A, CC2D1B, ADAM28, LRRC69, CEACAM1, THEM4 | LCK, TLR4 |
| 5 | hsa-miR-130b-3p | 8 | 19 | SLC16A1-AS1, C097376.2, SLC26A4-AS1, NUTM2A-AS1, NEAT1, AC127502.2, AC105036.3, AP005131.7 | BRAF, GOLGA8A, MAP2K6, DPF2, KIDINS220, PPIL2, IGF2R, RNF24, RNASEL, PEX26, ZNF333, PXK, TMEM106A, NMT2, RPS27L, TMEM104, ZRANB2, NMNAT1, HNRNPR, | - |
| 6 | hsa-miR-140-3p | 4 | 33 | FAM198B-AS1, NEAT1, TMEM147-AS1, LINC00894 | FAF1, HMCES, CD59, ATP11B, RC3H2, MAP2K6, CDS2, CKAP4, MAPK13, CLEC7A, PEDS1, POLR2E, WSB1, GNL1, LIN7A, IGF2R, HIF1AN, RNF24, ITM2C, CSNK2A1, SLC31A1, FBXO34, SULT1B1, UGGT1, PTPMT1, SLC41A1, AHCYL2, SLC25A25, PI4K2A, NPRL3,  GRAP2, ADAM28, THEM4 | POLR2E, GRAP2 |
| 7 | hsa-miR-17-5p | 8 | 43 | AC097376.2, RFX3-AS1, NUTM2A-AS1, RPARP-AS1, NEAT1, LINC02086, SNHG16, AC110597.1 | TLK2, HPSE, TRMT2A, AMN1, STT3A, SIPA1L1, PHF19, CCR6, FBXO30, DEDD, WSB1, TLR4, FBXO34, PEDS1, CERS6, SUGP2, ADAM28, PDAP1, WDFY3, DDHD2, NIPSNAP3A, ABHD5, DCTN4, HIF1AN, PEX26, UGGT1, SPCS3, MTARC1, NMNAT1, KRI1, SLC35E1, MAPK13, LIN7A, SPTLC2, DPF2, FAF1, BRAF, CSNK2A1, ATP8B1, DPH2, CEACAM1, CYP4F2, TCF7 | TLR4 |
| 8 | hsa-miR-181a-5p | 5 | 19 | AC239868.1, HCG11, AC009902.3, NEAT1, AC008543.1 | CD59, MAP2K6, APH1B, CLEC7A, KIDINS220, GNL1, CYTH2, RNF24, NUDT1, RIPK1, ATXN3, ABHD4, GRB10, IL15RA, BLNK, HAPLN3, PTPRE, SPG7, CEACAM1 | RIPK1 |
| 9 | hsa-miR-18a-5p | 1 | 31 | NEAT1 | GSE1, TLK2, TMEM106A, ZNF333, COA8, REEP4, WSB1, PPIL2, CSNK2A1, SPG7, CD59, CALHM2, SUGP2, HECW2, TOMM70, RPS27L, ABHD5, EFHC1, CSNK1A1, MAP2K6, ATXN3, TOM1, FAF1, TUT1, CEACAM1, RC3H2, PEX26,  TCF7, ECSIT, SCRN1, MR1 | - |
| 10 | hsa-miR-19b-3p | 5 | 6 | SLC16A1-AS1, PURPL, SLC26A4-AS1, NEAT1, AC005394.2 | TLR5, HECW2, ARFGEF1, PEX26, ATXN3, USP24 | - |
| 11 | hsa-miR-20a-5p | 8 | 20 | AC097376.2, RFX3-AS1, NUTM2A-AS1, RPARP-AS1, NEAT1, LINC02086, SNHG16, AC110597.1 | TLK2, STT3A, RNF24, FKBP9, CCR6, PTPRE, WSB1, TLR4, TCF7, HECW2, DCTN4, UGGT1, KRI1, LIN7A, KIDINS220, BRAF, DPH2, ATP6V1C1, MYO1C, PEX26 | TLR4 |
| 12 | hsa-miR-21-5p | 6 | 10 | AC000120.1, FAM201A, NUTM2A-AS1, F111167.2, CARD8-AS1, BRWD1-IT1 | CCR7, PELP1, APH1B, CSNK2A1, SPOCK2, DDHD2, SEC31B, RNF24, KIDNS220, PHETA1 | CCR7 |
| 13 | hsa-miR-26b-5p | 9 | 4 | AC239868.1, HCG11, AC000120.1, RPARP-AS1, NEAT1, AC127502.2, AC016876.2, AC015726.1, DLGAP1-AS5 | REEP4, ABTB1, MAP2K6, HPSE | - |
| 14 | hsa-miR-652-3p | 2 | 35 | AC006017.1, NEAT1 | PPIL2, SLC25A25, STT3A, CBR1, TAFAZZIN, GPS1, THEM4, PTPRE, DAZAP1, ZNF776, PTPMT1, PEDS1, TCF7, SPG7, SPOCK2, TLR5, DDHD2, PDAP1, PPP1R13B, HIF1AN, PEX26, FBXO34, PI4K2A, HMCES, ABHD4, MAPK13, MAP2K6, RIPK1, GNL1, BRAF, ABHD5, MR1, CD59, RNF24, ATXN3 | RIPK1 |
| 15 | hsa-miR-92a-3p | 6 | 54 | AC097376.2, PURPL, AC093627.4, LINC00858, LINC02321, AC005394.2 | SLCO4C1, FKBP9, HPCAL1, GON4L, TLK2, TMEM106A, PUS1, CCR6, DEDD, SLC25A25, FMNL2, PHETA1, ABTB1, GTF2IRD2, CLEC7A, FAM102A, CD59, GRB10, SPOCK2, RNF24, PPIL2, PELP1, SULT1B1, PDAP1, RPS27L, COX16, HIF1AN, PEX26, UGGT1,CRY2, MTARC1, KRI1, SLC31A1, CSNK2A1, POLR2E, MAPK13, MAP2K6, RIPK1, CAPS, MAP4K2, MED1, SPTLC2, DPF2, HPSE, DAZAP1, ATP8B1, POMT1, DPH2, MR1, LIG1, C21orf91, RC3H2, DCTN4, ATXN3 | POLR2E, RIPK1 |
| Abbreviations: DEmiRNAs, differentially expressed miRNAs; DETmRNA: differentially expressed target mRNA; DETlncRNAs: differentially expressed target lncRNA; PPI: Protein-Protein Interaction. | | | | | | |
