# Supplementary material for: Competing endogenous RNA network analysis of the molecular mechanisms of ischemic stroke
Source: BMC Genomics. 2023 Feb 8;24:67. doi: 10.1186/s12864-023-09163-1 (PMC9906963; doi:10.1186/s12864-023-09163-1)
Supplement: Supplementary file 4 — Additional file 4. ROC curve of key miRNAs in the validation set:(a) has-miR-140-3p; (b) has-miR-103a-3p; (c) has-miR-17-5p; (d) has-miR-18a-5p; (e) has-let-5d-5p; (f) has-miR-7f-5p; (g) has-miR-652-3p; (h) has-miR-92a-3p. [file 12864_2023_9163_MOESM4_ESM.pdf]

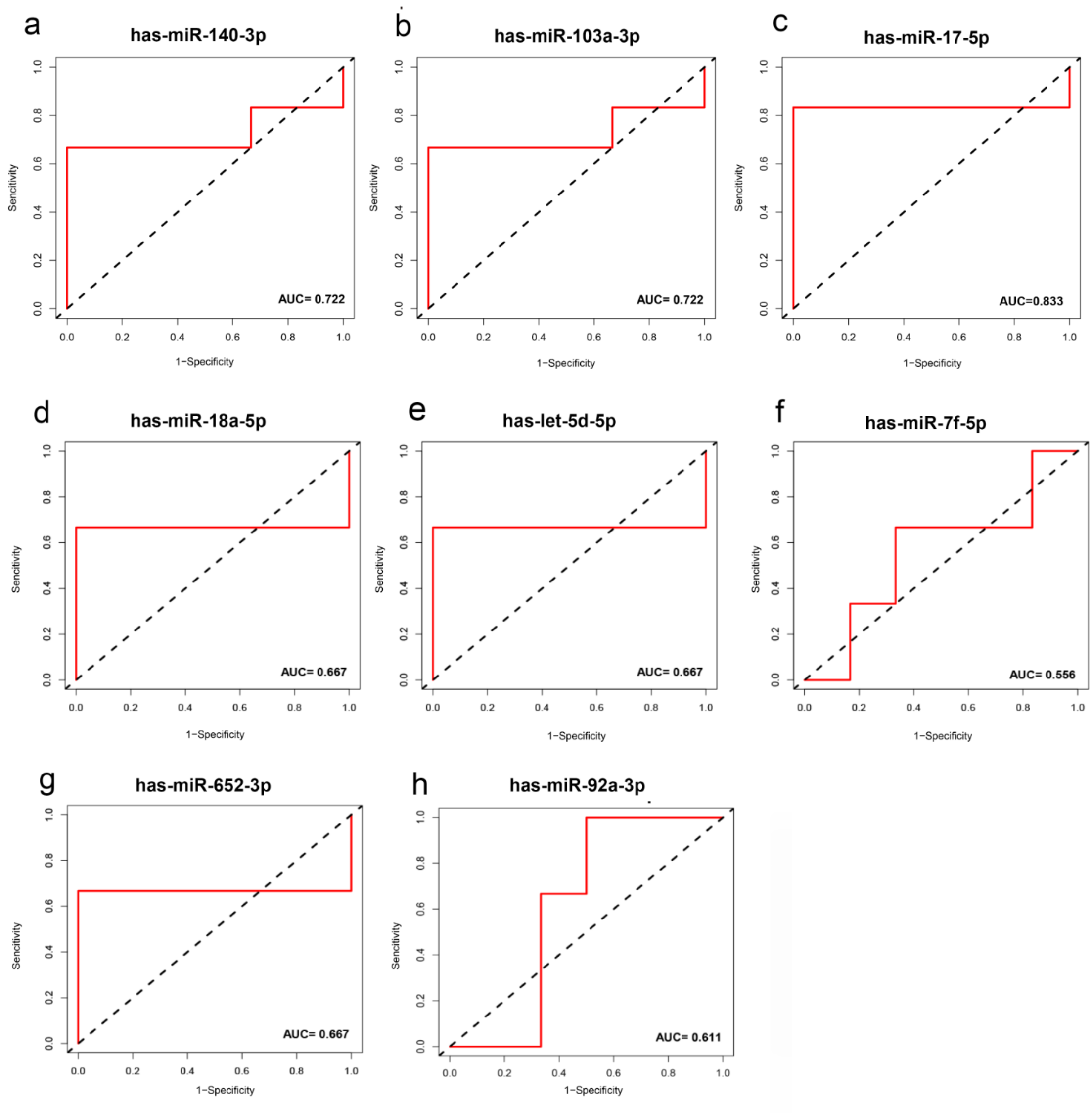

ROC curve of key miRNAs in the validation set：(a) has-miR-140-3p; (b) has-miR-103a-3p; (c) has-miR-17-5p; (d) has-miR-18a-5p; (e) has-let-5d-5p; (f) has-miR-7f-5p; (g) has-miR-652-3p; (h) has-miR-92a-3p.
